# Optimal treatment strategy and prognostic analysis for patients with locally advanced Upper Tract Urothelial Carcinoma

**DOI:** 10.3389/fsurg.2025.1548191

**Published:** 2025-06-02

**Authors:** Fan Jiang, Ruijie Dai, Mingguo Zhou, Xuefei Cao, Jun Lu, Xinyu Zheng

**Affiliations:** ^1^ Department of Surgery, Honghui Hospital, Xi’an Jiaotong University, Xi’an, China; ^2^ Department of Urology, First Affiliated Hospital of Xi’an Jiaotong University, Xi’an, China

**Keywords:** UTUC, SEER, T3-4M0, N0/+, prognosis, treatment

## Abstract

**Objective:**

This study aims to identify the optimal treatment strategy and conduct a prognostic analysis for patients with locally advanced Upper Tract Urothelial Carcinoma (UTUC).

**Methods and materials:**

The study included 3,829 patients diagnosed with pT3-4N0/+M0 UTUC from 2004 to 2015, with data obtained from the Surveillance, Epidemiology, and End Results (SEER) database. Patients were randomly assigned to a training group (70%) and a validation group (30%) for nomogram development. Variables that were significant in univariate COX regression analysis (*P* < 0.05) were included in the multivariate COX regression model, and a nomogram was formulated based on the variables that remained statistically significant (*P* < 0.05) in the multivariate analysis. The nomogram's predictive precision and ability to differentiate were evaluated through the concordance index(C-index), area under the curve (AUC), and calibration curves. The model's clinical validity was confirmed through the use of decision curve analysis (DCA).

**Results:**

Within the pN+ subgroup, the combination of surgery with both adjuvant chemotherapy and radiotherapy (S + R + C) group and S + C group yielded superior results over the S group, with the S + R + C group regimen showing the most favorable outcomes. The 3-year OS rates for patients in the S + R + C, S + C, and S groups were recorded as 40.00%, 31.43%, and 12.5%. The corresponding 3-year CSS rates were 47.56%, 34.02%, and 17.5%. Multivariate COX regression analysis identified age, primary tumor location, T and N stages, treatment modality, tumor size, and lymph node count as significant predictors of OS and CSS. These factors were integrated into precisely developed nomograms for predicting OS and CSS, with concordance indices of 0.651 and 0.667 in both sets.

**Conclusion:**

For patients with pT3-4N + M0 stage UTUC, the addition of radiotherapy to the surgical and chemotherapy regimen has proven to enhance survival rates. Our predictive nomogram reliably forecasts OS and CSS rates for locally advanced patients. This tool can assist clinicians in identifying high-risk individuals, thereby aiding in the formulation of informed treatment decisions.

## 1 Introduction

Upper Tract Urothelial Carcinoma (UTUC) is a rare malignant tumor of the urinary system, arising from the renal pelvis and ureter. It represents around 5%–10% of all urothelial cancers (1, 2). In Western nations, the annual occurrence is roughly 2 per 100,000 individuals. The incidence is higher in East Asia, possibly due to the consumption of traditional Chinese medicinal herbs containing aristolochic acid (3). Locally advanced UTUC, classified as T3-4N0/+M0 staging, constitutes 12.6% of cases and is associated with a poorer prognosis (4). Radical nephroureterectomy (RNU) with bladder cuff excision is the standard treatment for these patients. Most experts agree that postoperative chemotherapy with platinum agents improves outcomes for locally advanced UTUC patients (5–10). Recent systematic comparisons of UTUC guidelines have highlighted significant discrepancies in treatment protocols, particularly regarding the application of adjuvant chemotherapy and radiotherapy for locally advanced cases (11). These findings underscore the absence of unified evidence-based guidelines, which in turn necessitates further exploration of optimal treatment sequencing. Specifically, the role of adjuvant radiotherapy after UTUC surgery remains a contentious issue, with its added value compared to chemotherapy still being questioned (12).

Our retrospective analysis used the Surveillance, Epidemiology, and End Results (SEER) database to assess the survival statistics among individuals with locally advanced UTUC treated with various therapeutic approaches. Our aim was to identify prognostic factors and develop validated predictive models for overall survival (OS) and cancer-specific survival (CSS). By analyzing a large cohort of pT3-4N0/+M0 UTUC patients, this study seeks to clarify the survival benefits of surgery combined with adjuvant chemotherapy or chemoradiotherapy, thereby providing more consistent therapeutic guidance for clinical practice.

## 2 Materials and methods

### 2.1 Study population

**Figure 1:**
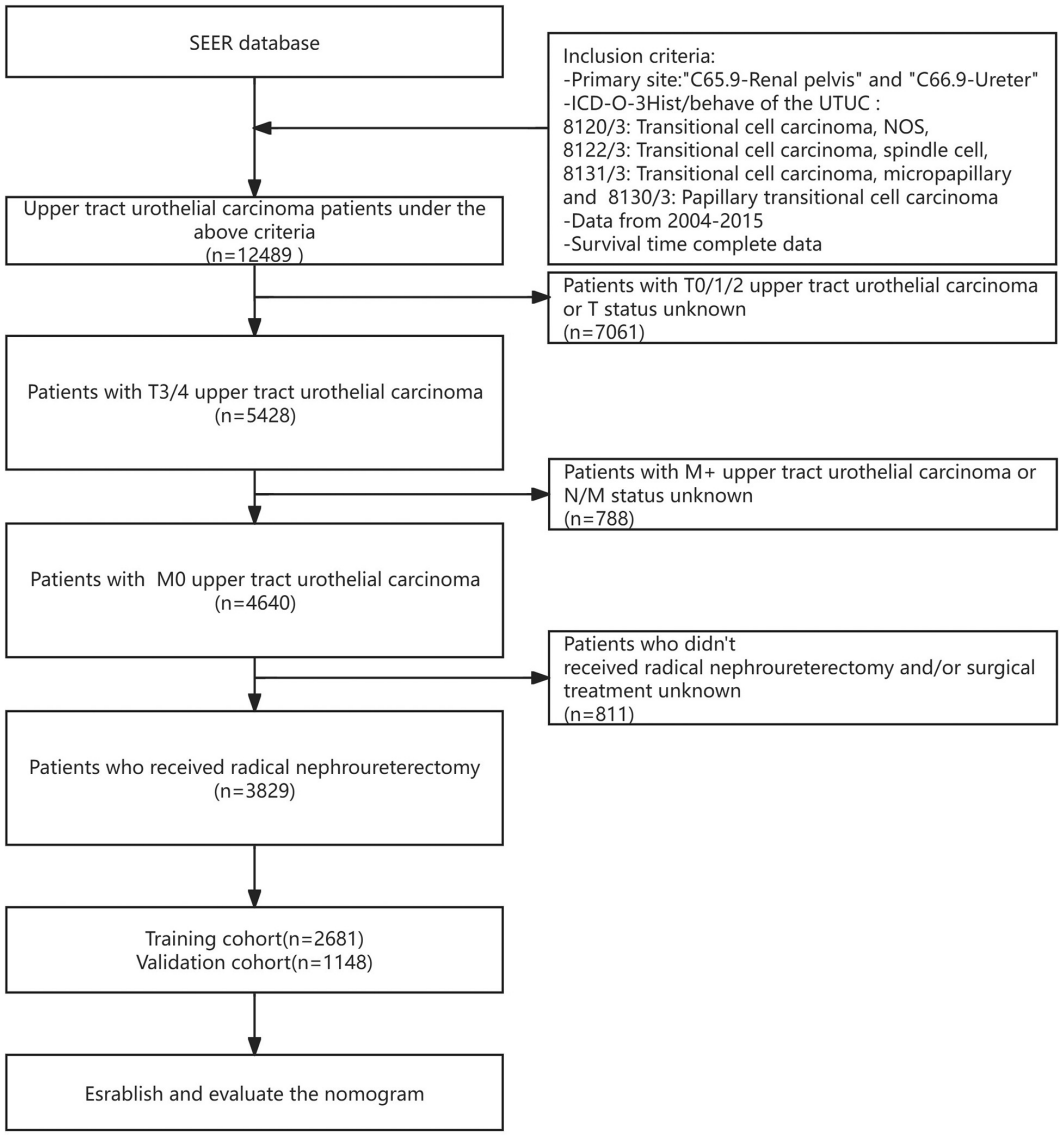
Flowchart of screening process.

For our analysis using the SEER database, the following criteria were applied to include patients: (1) diagnosis of primary UTUC between January 2004 and December 2015; (2) histological type of transitional cell carcinoma, identified by ICD-O-3 codes 8120/3, 8122/3 and 8130/3; (3) availability of complete survival data; (4) AJCC classification of T3-4 M0; (5) Detailed records, including cause of death and specifics of treatments such as surgery, radiation, and chemotherapy, were required for inclusion. The final study population included 3,829 individuals with stage pT3-4 M0 disease, all of whom received surgery as part of their initial treatment regimen. The specific process is depicted in Figure 1.

### 2.2 Treatment groups

Each patient in the study underwent surgical treatment. The individuals who underwent surgery exclusively were placed into the S group. Patients were categorized into the S + R group if they received postoperative radiotherapy and into the S + C group if they underwent postoperative chemotherapy. Those treated with a combination of chemotherapy and radiotherapy post-surgery were placed in S + R + C Group.

### 2.3 Statistical analysis

The research study defined overall survival (OS) as the period from the point of randomization to the time of death due to any reason. Cancer-specific survival (CSS) was characterized as the time from randomization to death caused by UTUC. OS was chosen as the main focus, while CSS was identified as the secondary measure.

Analyses were performed using R Software version 4.3.1 and SPSS 26.0, with graphical representations generated through GraphPad Prism 8 and multiple R packages, such as “timeROC”, “rms”, “survival”, “timeROC”, “ggplot2”, “dplyr”, “foreign”, “dcurves” and “nomogramFormula”. Patients were randomly allocated to the training and validation cohorts in a 7:3 ratio using propensity score matching (PSM) to minimize selection bias. Matching variables included age, sex, race, marital status, primary tumor site, T stage, N stage, radiation status, chemotherapy status, treatment modality, number of regional lymph nodes removed, and tumor size. Baseline characteristics between the two cohorts were compared using Pearson's chi-square test, confirming balanced distributions across all matched variables (Table 1).

**Table 1:** Baseline characteristics of included patients.

Characteristics	Overall	Training cohort	Validation cohort	*P*-value
	*N* = 3,829 no. (%)	*N* = 2,681 no. (%)	*N* = 1,148 no. (%)	
Age				
<57	342 (8.9)	241 (9.0)	101 (8.8)	0.918
57–77	2,122 (55.4)	1,480 (55.2)	642 (55.9)	
>77	1,365 (35.6)	960 (35.8)	405 (35.3)	
Sex				
Female	1,558 (40.7)	1,100 (41.0)	458 (39.9)	0.536
Male	2,271 (59.3)	1,581 (59.0)	690 (60.1)	
Race				
White	3,379 (88.2)	2,375 (88.6)	1,004 (87.5)	0.593
Black	156 (4.1)	105 (3.9)	51 (4.4)	
Other	294 (7.7)	201 (7.5)	93 (8.1)	
Marriage				
Divorced/Single/Widowed/Separated	1,384 (36.1)	969 (36.1)	415 (36.1)	0.713
Married	2,325 (60.7)	1,632 (60.9)	693 (60.4)	
Unknow	120 (3.1)	80 (3.0)	40 (3.5)	
Primary site				
Renal pelvis	2,882 (75.3)	2,030 (75.7)	852 (74.2)	0.344
Ureter	947 (24.7)	651 (24.3)	296 (25.8)	
T stage				
T3	3,194 (83.4)	2,240 (83.6)	954 (83.1)	0.768
T4	635 (16.6)	441 (16.4)	194 (16.9)	
N stage				
N0	3,063 (80.0)	2,137 (79.7)	926 (80.7)	0.162
N1	381 (10.0)	272 (10.1)	109 (9.5)	
N2	306 (8.0)	224 (8.4)	82 (7.1)	
Nx	79 (2.1)	48 (1.8)	31 (2.7)	
Radiation				
No/Unknow	3,575 (93.4)	2,510 (93.6)	1,065 (92.8)	0.368
Yes	254 (6.6)	171 (6.4)	83 (7.2)	
Chemotherapy				
No/Unknow	2,783 (72.7)	1,944 (72.5)	839 (73.1)	0.745
Yes	1,046 (27.3)	737 (27.5)	309 (26.9)	
Treatment				
S	2,678 (69.9)	1,880 (70.1)	798 (69.5)	0.22
S + R	105 (2.7)	64 (2.4)	41 (3.6)	
S + C	897 (23.4)	630 (23.5)	267 (23.3)	
S + R+C	149 (3.9)	107 (4.0)	42 (3.7)	
Regional lymph nodes removed				
0	2,487 (65.0)	1,725 (64.3)	762 (66.4)	0.374
1–3	716 (18.7)	516 (19.2)	200 (17.4)	
4 or more	555 (14.5)	394 (14.7)	161 (14.0)	
Unknow	71 (1.9)	46 (1.7)	25 (2.2)	
Size				
1–28	857 (22.4)	599 (22.3)	258 (22.5)	0.992
29–39	711 (18.6)	495 (18.5)	216 (18.8)	
40–59	956 (25.0)	672 (25.1)	284 (24.7)	
>59	940 (24.5)	656 (24.5)	284 (24.7)	
Unknow	365 (9.5)	259 (9.7)	106 (9.2)	

Survival data were assessed using the Kaplan–Meier method and compared via the log-rank test. Significant variables from univariate analysis (*P* < 0.05) were included in a multivariable Cox regression model using forward stepwise selection. Variables retaining independent prognostic significance (*P* < 0.05) were incorporated into the nomogram.

The nomogram's predictive accuracy was evaluated through the concordance index (C-index), ROC curves, and calibration plots. Decision curve analysis (DCA) was implemented to assess clinical utility and benefits. X-tile software version 3.6.1 from Yale University was used to identify cut-off values for both patient age and tumor size, as well as for the predictive model's nomogram scores which were utilized to stratify patients into low-, moderate-, and high-risk groups.

## 3 Results

### 3.1 Characteristics of the patient

From the SEER database, we identified 12,489 individuals with a diagnosis of UTUC between January 2004 and December 2015. Exclusions were made for 7,061 patients with stages T0-T2, 788 with metastatic or indeterminate N/M classification, and 811 who did not undergo radical nephroureterectomy, narrowing down the study population to 3,829 patients. These subjects were assigned randomly to either a training group or a validation group in a 7:3 ratio, with important traits detailed in Table 1.

**Figure 2:**
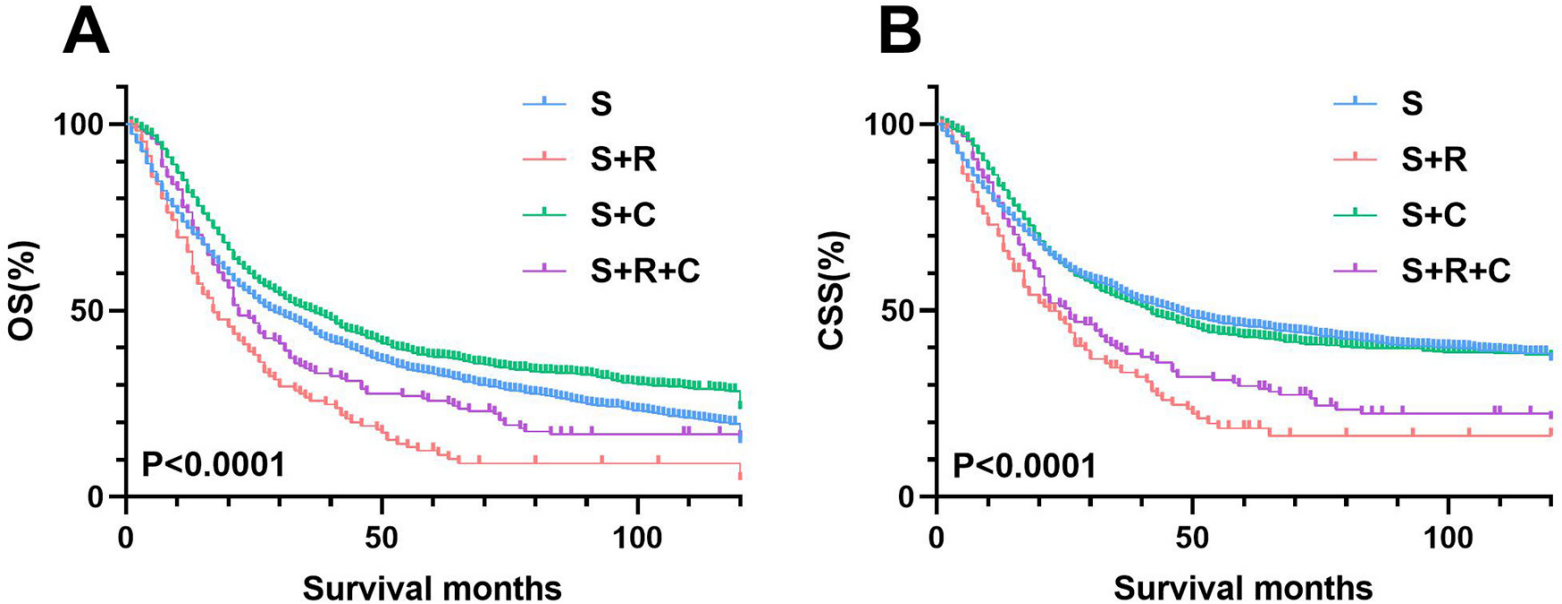
Cohort analysis of survival rates in T3-4M0 UTUC patients by treatment type. **(A)** OS rates between four groups **(B)** OS rates between S and S+ ADT groups.

The median survival for the entire SEER cohort was 29 months, with 1-, 3-, and 5-year overall OS rates of 74.31%, 45.00%, and 33.19%, respectively, and CSS rates of 79.02%, 53.16%, and 43.71%. The S + C group's OS and CSS rates either matched or exceeded those of the S group. In contrast, survival rates for patients treated with postoperative radiotherapy (S + R or S + R + C) were consistently inferior to the S group, highlighting the limited clinical benefit of radiotherapy in this cohort (Figure 2).

### 3.2 Independent prognostic predictors of OS and CSS

In the training group's univariate COX regression, significant predictors for OS and CSS included age, tumor location, T and N stages, treatment method, tumor size, and number of lymph nodes removed (Table 2). After adjusting for covariates in the multivariate COX regression analysis, the independent prognostic factors for OS and CSS were confirmed to include age, primary tumor location, T stage, N stage, treatment strategy, number of regional lymph nodes removed, and tumor size (Table 3).

**Table 2:** Univariate COX regression analysis of OS and CSS.

Variable	Univariate COX regression analysis					
	OS			CSS		
	HR	95% CI	*P*	HR	95% CI	*P*
Age						
<57						
57–77	1.939	1.608–2.339	0.000	1.562	1.275–1.913	0.000
>77	3.223	2.669–3.907	0.000	2.098	1.703–2.584	0.000
Sex						
Female						
Male	0.950	0.871–1.037	0.25	0.952	0.858–1.056	0.352
Primary site						
Renal pelvis						
Ureter	1.188	1.077–1.310	0.000	1.243	1.108–1.395	0.000
T stage						
T3						
T4	2.013	1.803–2.247	0.000	2.321	2.049–2.628	0.000
N stage						
N0						
N1	1.693	1.475–1.943	0.000	2.134	1.833–2.483	0.000
N2	1.916	1.653–2.221	0.000	2.340	1.988–2.756	0.000
Nx	1.471	1.075–2.013	0.016	1.632	1.135–2.346	0.008
Radiation						
No/Unknow						
Yes	1.340	1.133–1.586	0.000	1.597	1.329–1.919	0.000
Chemotherapy						
No/Unknow						
Yes	0.832	0.754–0.918	0.000	1.064	0.952–1.189	0.273
Treatment						
S						
S + R	1.474	1.136–1.912	0.004	1.671	1.243–2.247	0.001
S + C	0.800	0.719–0.889	0.000	1.017	0.902–1.146	0.789
S + R + C	1.159	0.934–1.437	0.180	1.568	1.246–1.972	0.000
Regional lymph nodes removed						
0						
1 to 3	1.162	0.041–1.297	0.008	1.195	1.048–1.363	0.008
4 or more	0.993	0.875–1.127	0.911	1.129	0.976–1.306	0.103
Unknow	1.530	1.121–2.087	0.007	1.714	1.205–2.439	0.003
Size						
1–28						
29–39	1.237	1.077–1.422	0.003	1.328	1.120–1.575	0.001
40–59	1.327	1.168–1.509	0.000	1.433	1.224–1.679	0.000
>59	1.794	1.579–2.038	0.000	2.140	1.835–2.496	0.000
Unknow	1.586	1.348–1.867	0.000	1.733	1.422–2.112	0.000

**Table 3:** Multivariate COX regression analysis of OS and CSS.

Variable	Multivariate COX regression analysis					
	OS			CSS		
	HR	95% CI	*P*	HR	95% CI	*P*
Age						
<57						
57–77	1.995	1.652–2.410	0.000	1.662	1.354–2.041	0.000
>77	3.330	2.739–4.047	0.000	2.348	1.894–2.912	0.000
Primary. Site						
Renal pelvis						
Ureter	1.261	1.138–1.397	0.000	1.361	1.206–1.535	0.000
T Stage						
T3						
T4	1.694	1.509–1.902	0.000	1.826	1.601–2.083	0.000
N Stage						
N0						
N1	1.999	1.705–2.344	0.000	2.370	1.981–2.836	0.000
N2	2.400	2.011–2.863	0.000	2.658	2.174–3.250	0.000
NX	1.397	1.017–1.920	0.039	1.511	1.046–2.182	0.028
Treatment						
S						
S + R	1.211	0.931–1.574	0.154	1.356	1.006–1.827	0.045
S + C	0.796	0.709–0.893	0.000	0.908	0.796–1.036	0.153
S + R + C	1.004	0.802–1.257	0.974	1.185	0.931–1.508	0.169
Regional lymph nodes removed						
0						
1–3	0.813	0.717–0.922	0.001	0.745	0.640–0.867	0.000
4 or more	0.707	0.611–0.817	0.000	0.690	0.581–0.818	0.000
Unknow	1.100	0.797–1.519	0.561	0.997	0.690–1.441	0.988
Size						
1–28						
29–39	1.251	1.087–1.439	0.002	1.356	1.142–1.611	0.000
40–59	1.286	1.129–1.464	0.000	1.398	1.190–1.641	0.000
59-	1.658	1.450–1.897	0.000	1.903	1.617–2.238	0.000
Unknow	1.385	1.172–1.637	0.000	1.471	1.201–1.801	0.000

### 3.3 Construction and validation of the nomogram

**Figure 3:**
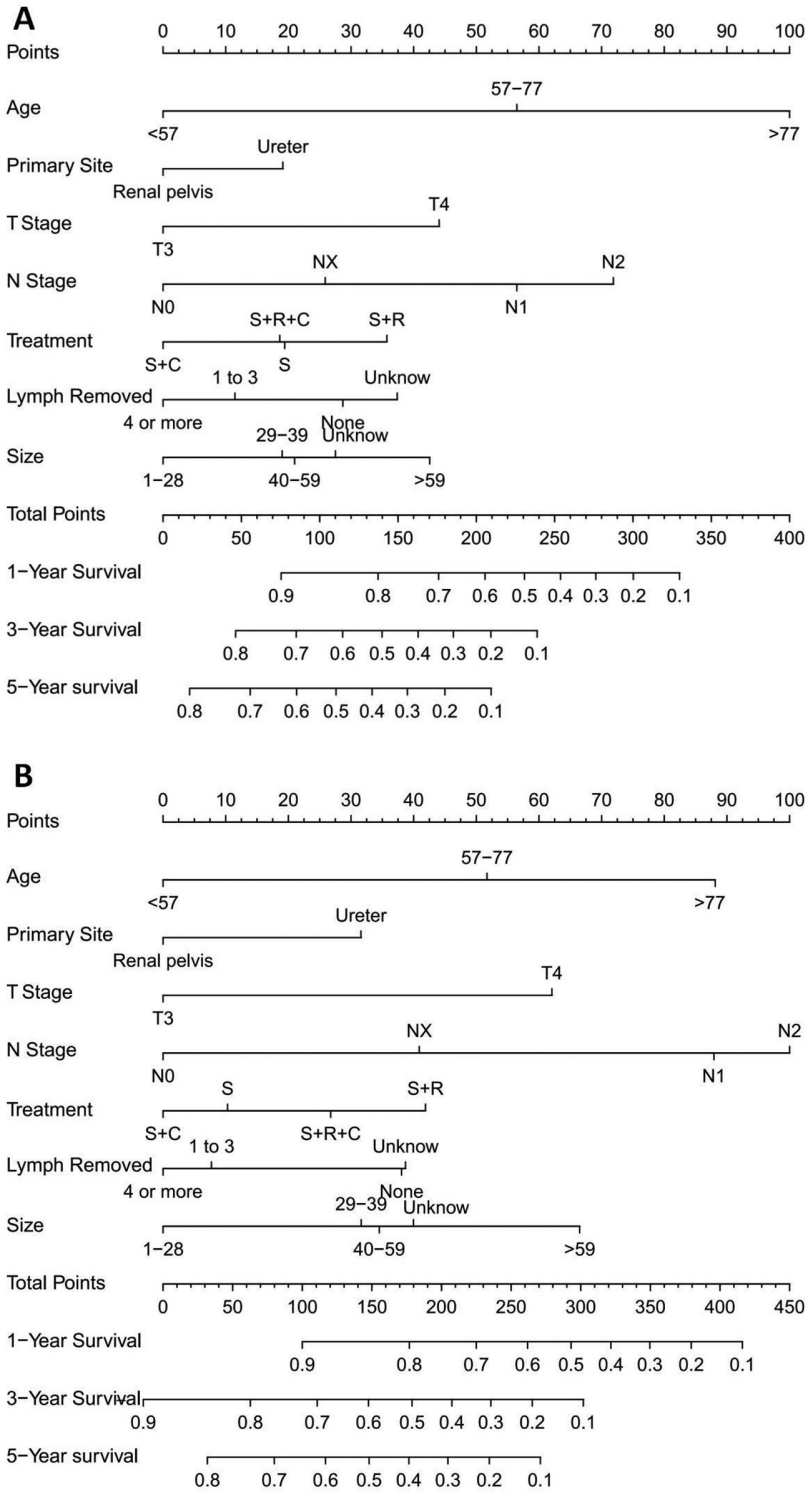
Prognostic nomograms for 1-, 3-, and 5-year OS and CSS in T3-4M0 UTUC patients. **(A)** Nomogram for predicting 1-, 3-, and 5-year OS; **(B)** Nomogram for predicting 1-, 3-, and 5-year CSS.

The nomogram for predicting OS and CSS was developed based on the prognostic factors identified through the multivariate COX regression analysis of the training group (Figures 3A,B). Age emerged as the primary determinant for OS, followed by N stage, T stage, tumor size, number of lymph node removal, treatment approach, and primary tumor location. For CSS, N stage was the primary predictor, succeeded by age, tumor size, T stage, treatment strategy, number of regional lymph nodes removed and primary tumor location.

**Figure 4:**
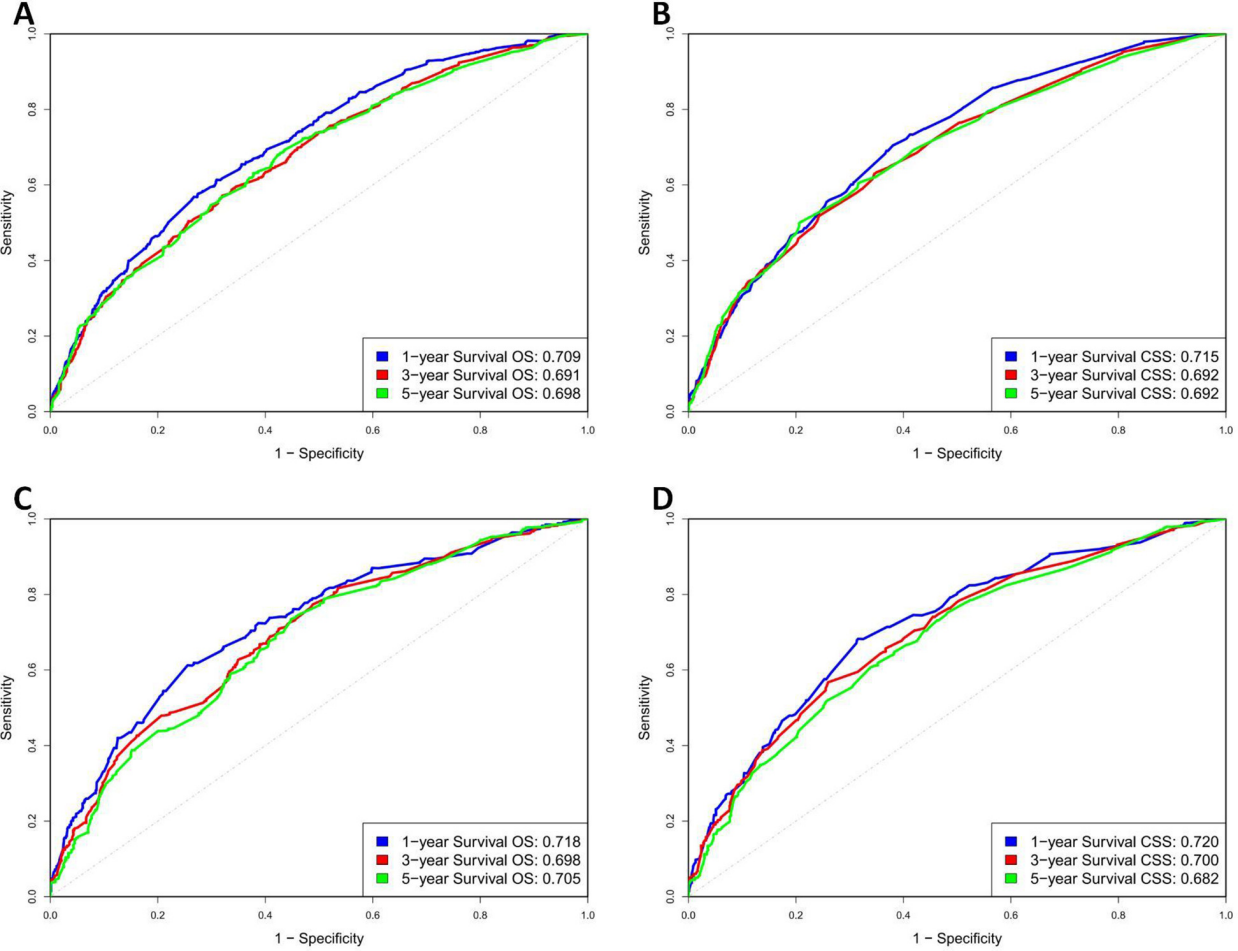
ROC curve of OS and CSS in training and validation cohorts. **(A)** Training group OS; **(B)** Training group CSS; **(C)** Validation group OS; **(D)** Validation group CSS.

In the training group, the C-index values for predicting OS and CSS were 0.651 and 0.667, respectively, which increased to 0.666 and 0.669 in the validation group, indicating good predictive accuracy. The 1-, 3-, and 5-year OS AUCs for the training group were 0.709, 0.691, and 0.698, respectively, while the validation group showed slightly higher values of 0.718, 0.698, and 0.705 (Figures 4A,C). For CSS, the training group's AUCs were 0.715, 0.692, and 0.692, and the validation group exhibited AUCs of 0.720, 0.700, and 0.682, respectively (Figures 4B,D).

**Figure 5:**
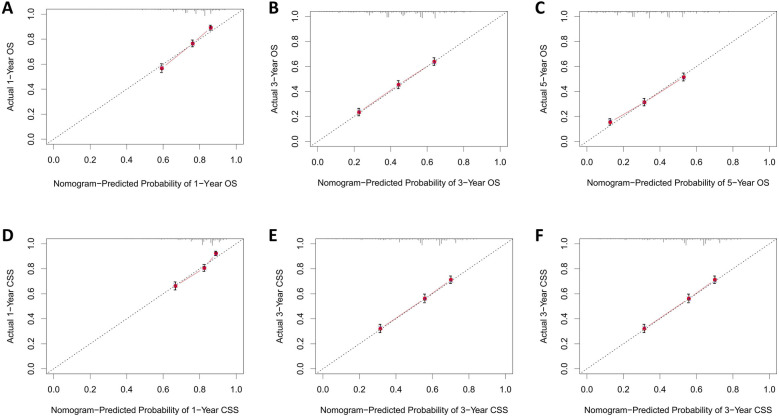
Calibration curves of training group 1-, 3-, and 5-year OS and CSS. **(A)** Training group 1-year OS; **(B)** Training group 3-year OS; **(C)** Training group 5-year OS; **(D)** Training group 1-year CSS; **(E)** Training group 3-year CSS; **(F)** Training group 5-year CSS.

**Figure 6:**
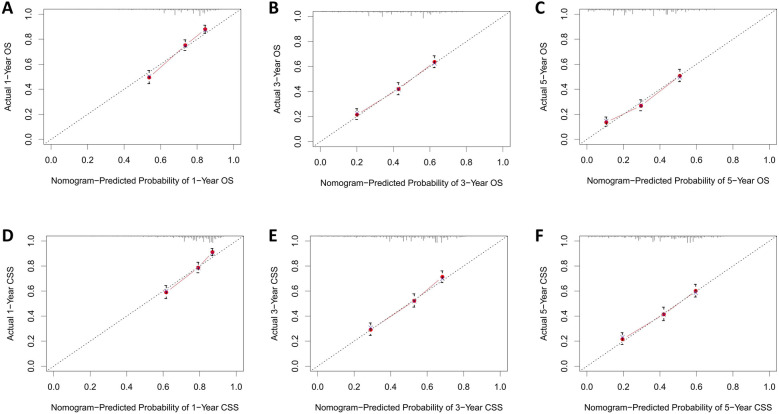
Calibration curves of validation group 1-, 3-, and 5-year OS and CSS. **(A)** Validation group 1-year OS; **(B)** Training group 3-year OS; **(C)** Validation group 5-year OS; **(D)** Validation group 1-year CSS; **(E)** Validation group 3-year CSS; **(F)** Validation group 5-year CSS.

**Figure 7:**
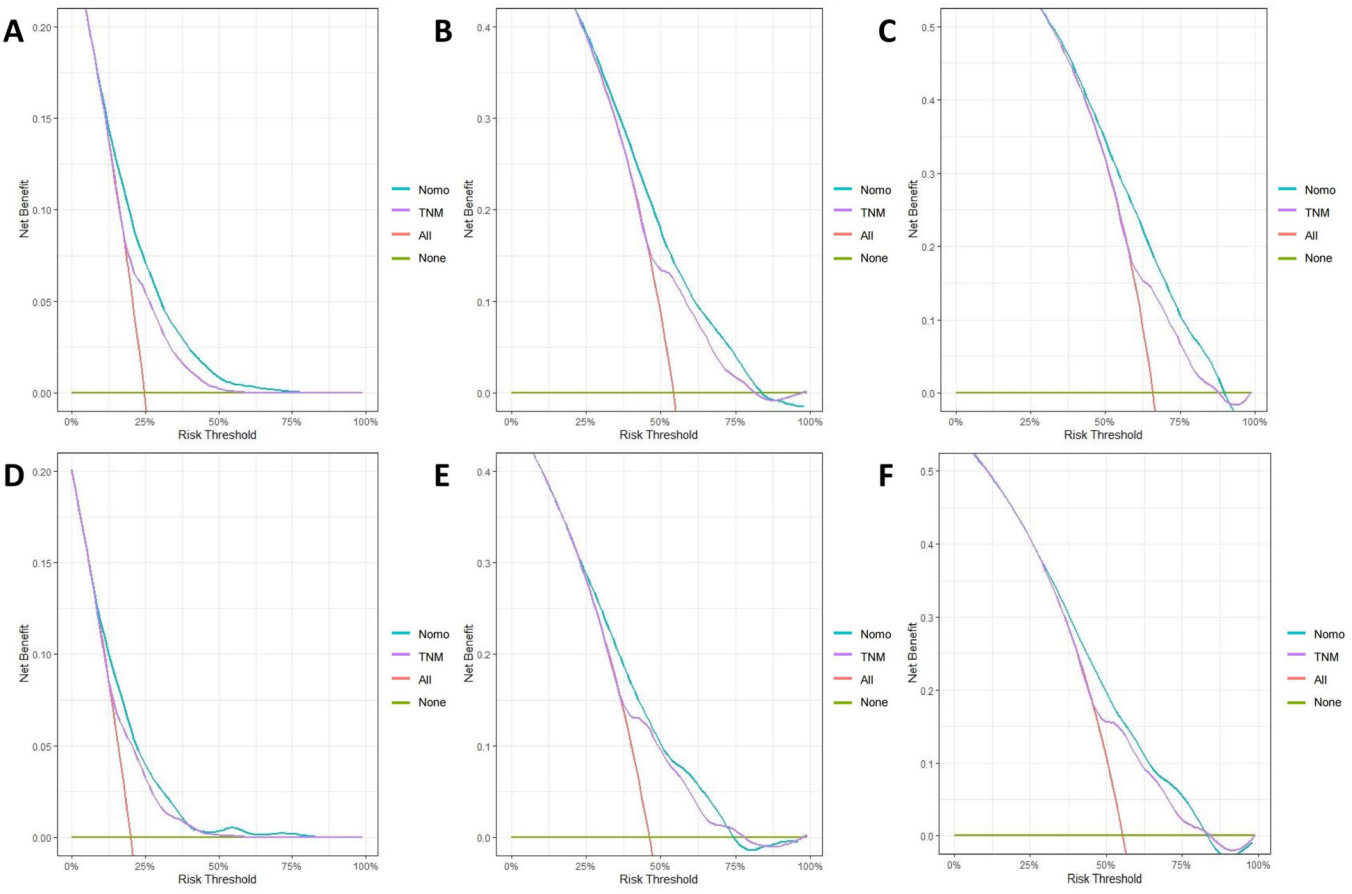
Decision curves of training group 1-, 3-, and 5-year OS and CSS **(A)** 1-year OS; **(B)** 3-year OS; **(C)** 5-year OS; **(D)** 1-year CSS; **(E)** 3-year CSS; **(F)** 5-year CSS.

**Figure 8:**
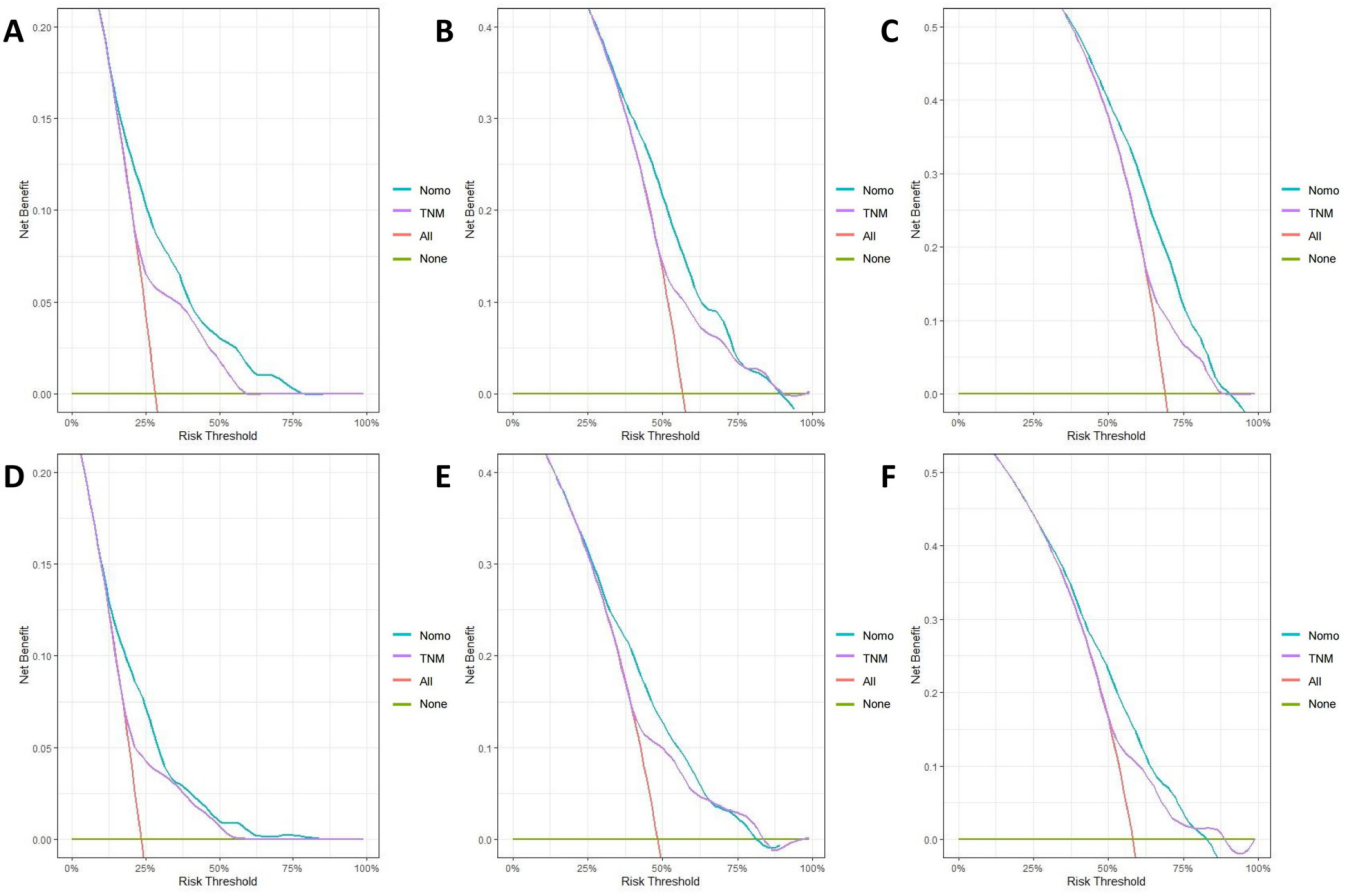
Decision curves of validation group 1-, 3-, and 5-year OS and CSS **(A)** 1-year OS; **(B)** 3-year OS; **(C)** 5-year OS; **(D)** 1-year CSS; **(E)** 3-year CSS; **(F)** 5-year CSS.

The calibration curves for both the training and validation groups showed excellent agreement between predicted and observed outcomes for 1-, 3-, and 5-year OS and CSS (Figures 5, 6). DCA validated the robust clinical applicability of the nomograms for forecasting 1-, 3-, and 5-year OS and CSS across both training and validation cohorts (Figures 7, 8).

**Figure 9:**
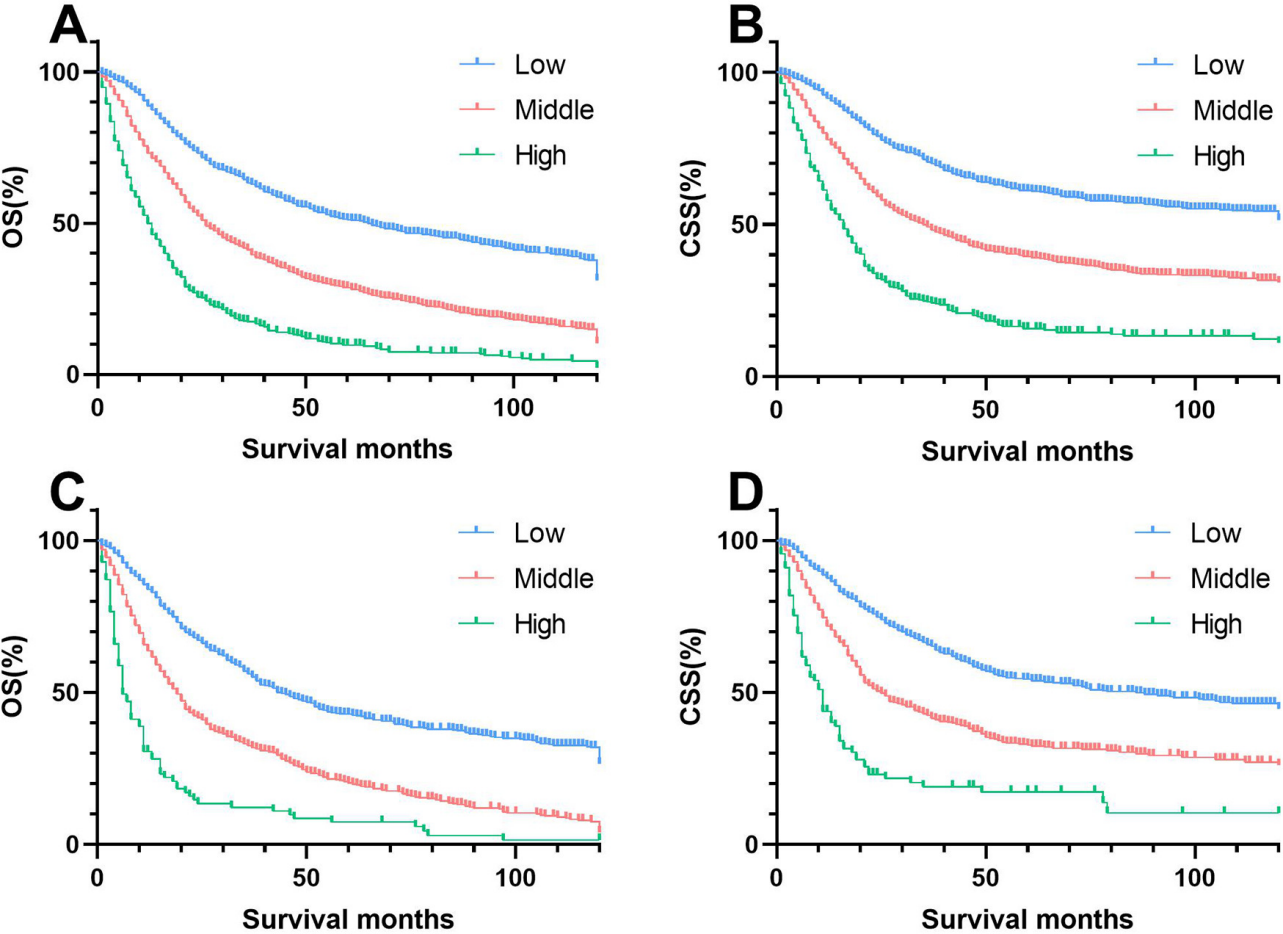
Kaplan–meier estimates of OS and CSS by risk group in training and validation cohorts. **(A)** Training group of OS; **(B)** Validation group of OS; **(C)** Training group of CSS; **(D)** Validation group of CSS.

In summary, the nomograms effectively stratified patients into three risk levels based on the risk scores calculated, using cut-off values determined by X-tile software. Patients were categorized into low risk (≤1101.47 points), intermediate risk (101.47 points to 166.72 points), and high risk (>166.72 points) using the OS nomogram. The CSS nomogram divided patients into low risk (≤94.78 points), intermediate risk (94.78 points to 167.14 points), and high risk (>167.14 points). Distinct variations in survival across risk subgroups were illustrated by Kaplan–Meier curves (Figure 9).

### 3.4 Subgroup analysis

**Figure 10:**
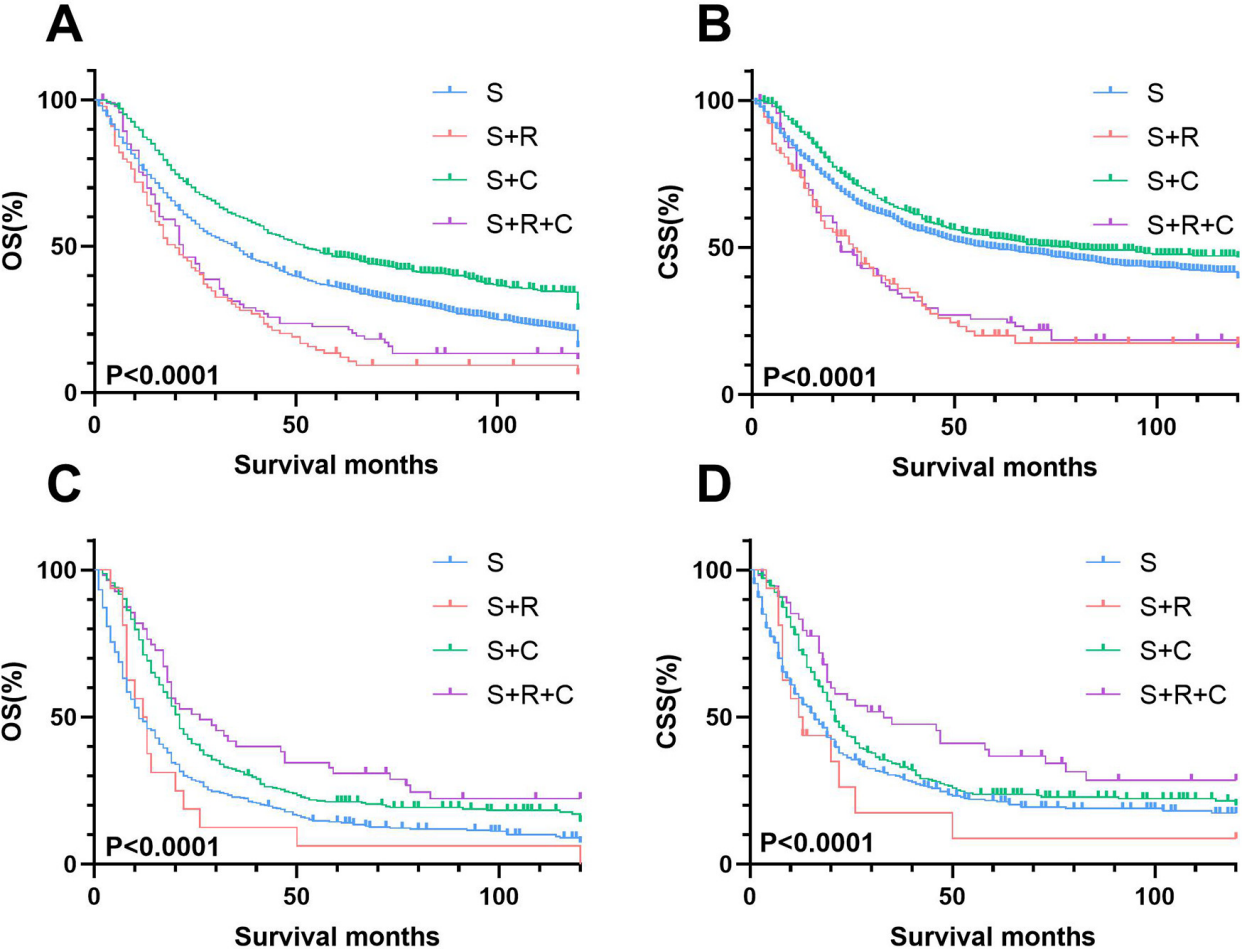
Stage-stratified kaplan–meier survival analysis for OS and CSS in T3-4N0M0 and T3-4N + M0 UTUC patients receiving different treatments. **(A)** T3-4N0M0 group of OS; **(B)** T3-4N0M0 group of CSS; **(C)** T3-4N + M0 group of OS; **(D)** T3-4N + M0 group of CSS.

Kaplan–Meier analysis of the pN+ subgroup demonstrated significantly improved survival outcomes for the S + R + C and S + C groups compared to the S group (*P* < 0.001), with the S + R + C regimen achieving the most favorable results (Figure 10). The S + R + C group exhibited superior 3-year OS (40.00% vs. 31.43% for S + C vs. 12.50% for S) and CSS (47.56% vs. 34.02% for S + C vs. 17.50% for S), with sustained benefits at 5 years (*P* < 0.001). In contrast, within the N0 subgroup, both S + R and S + R + C showed no significant survival advantage over S (3-year OS: 25.71% and 33.79% vs. 44.75%; 3-year CSS: 33.35% and 39.15% vs. 53.01%).These findings suggest that the addition of radiotherapy and chemotherapy may provide meaningful survival benefits for node-positive patients but may not be necessary for node-negative patients, potentially avoiding overtreatment.

Univariate and multivariate Cox regression analyses further confirmed these trends (Supplementary Tables S1, S2; Supplementary Figures S1, S2). While S + C consistently reduced mortality risk across all subgroups, S + R + C provided exclusive survival benefits in the pN+ cohort. These findings highlight the critical role of multimodal therapy (S + R + C) in pT3-4N + M0 UTUC, whereas adjuvant radiotherapy offered no clinical advantage in pN0 disease.

## 4 Discussion

Unlike previous SEER-based studies on UTUC, which predominantly focused on monotherapy efficacy and prior risk stratification models relied on conventional factors (e.g., T/N staging, age) but omitted therapeutic modalities and number of lymph node removal as prognostic variables (13–17). Our novel contribution lies in integrating multimodal treatment strategies (surgery ± chemotherapy/radiotherapy) into the prognostic framework, validated through analytical methods including competing risk models and propensity score matching and this study represents the first systematic evaluation of survival benefits conferred by combined radiotherapy in the pN+ subgroup. Importantly, the nomogram demonstrated robust stratification efficacy, outperforming existing models that lack therapeutic context (15, 18). This approach not only refines risk prediction but also underscores the necessity of tailoring adjuvant therapies to nodal status—a critical advancement in UTUC management.

For patients diagnosed with UTUC at stages T3-T4N0/+M0, radical nephroureterectomy (RNU) remains the cornerstone of primary treatment. Consistent with international consensus (5–10), The POUT study has shown that initiating a combination of gemcitabine and platinum chemotherapy shortly after nephroureterectomy can notably enhance disease-free survival (6). our findings reinforce that adjuvant chemotherapy significantly improves prognosis in locally advanced UTUC.

The role of radiotherapy (RT) as an adjuvant to surgery in UTUC remains controversial. While some studies suggest postoperative RT improves local control and survival in advanced UTUC (19–22), others report no benefit or even detrimental effects (12, 23–26). In our study, we observed that radiotherapy was actually a detrimental factor for prognosis across the entire cohort. The 1-, 3-, and 5-year OS rates for the S + R group were 65.71%, 25.71%, and 12.38%. For the S + R + C group, these rates were 76.36%, 33.79%, and 25.68%, respectively. The corresponding CSS rates were 70.03% and 78.75%, 33.35% and 39.15%, 18.38% and 29.75%, respectively. Their survival curves were consistently inferior to those of the S group. Subgroup analysis revealed that in both the N+ and N0 categories, the S + R group continued to be an unfavorable factor for prognosis.

However, a critical nuance emerged in the pT3-4N + M0 subgroup. Here, multimodal therapy (S + R + C) achieved superior 3-year OS (40.00% vs. 12.50% for S; *P* < 0.001) and CSS (47.56% vs. 17.50% for S; *P* < 0.001), outperforming both S + C (OS: 31.43%; CSS: 34.02%) and S alone. This suggests that RT, when combined with chemotherapy, may selectively benefit node-positive UTUC by targeting residual nodal micrometastases, whereas its utility in N0 disease remains unsubstantiated. Notably, this contrasts with bladder cancer literature, where Zaghloul et al. (27) reported improved locoregional control with adjuvant chemoradiotherapy, though its additive value to chemotherapy alone remains debated (12). The divergence may reflect anatomical and biological differences between UTUC and bladder cancer, such as thinner ureteral adventitia facilitating tumor spread (28) or distinct molecular drivers (15) This indicates that for pT3-4N + M0 UTUC patients, Multimodal therapy (S + R + C) should be prioritized, as it addresses both systemic (via chemotherapy) and locoregional (via RT) disease burdens, particularly in high-risk nodal metastases.

The selective efficacy of RT in N+ patients may stem from its ability to eradicate residual nodal disease after lymphadenectomy, a hypothesis supported by the prognostic significance of lymph node yield in our nomogram (HR: 0.71 for ≥4 nodes removed; *P* < 0.001). Nevertheless, the absence of standardized RT protocols (e.g., dose, field design) in UTUC limits cross-study comparisons and mandates prospective validation (19, 22).

Recent studies on the development of UTUC suggest that the most crucial phase for progression mainly takes place during the initial three years following the surgical procedure (19, 22). Our research supports these findings, demonstrating a 45.00% 3-year OS rate and a 53.16% 3-year CSS rate for individuals diagnosed with pT3-4M0 UTUC.

The factors influencing the prognosis for UTUC are still uncertain, with a multitude of patient- and tumor-related factors identified, such as age, genetic susceptibility, tobacco consumption, surgical delay, tumor stage and grade, tumor location and size, pathological subtypes, lymph node metastasis, surgical margin status, and lymphovascular invasion (15, 29–31). These factors can be utilized to stratify patients' risk and determine the most appropriate local treatment approach—radical vs. conservative—as well as to discuss perioperative systemic therapy.

Nomograms were created using multivariate COX regression analysis to forecast OS and CSS, taking into account variables including age, primary tumor location, T and N stages, treatment approach, tumor size and the number of lymph nodes removed.Validation was conducted by utilizing calibration curves, ROC curves, and DCA, along with additional verification from an independent validation cohort.

Our research indicates that age significantly influences survival outcomes in a manner independent of other factors. In the complete SEER cohort, 55.4% of patients were within the 57–77 age range, and 35.6% were 78 years or older. Compared to the youngest age group (≤56 years), older age groups demonstrated significantly higher mortality risks, with a hazard ratio (HR) of 1.911 for the 57–77 age group (95% CI: 1.633–2.233, *P* < 0.001), and a more pronounced HR of 3.299 for individuals over 78 years (95% CI: 2.816–3.866, *P* < 0.001). The elevated risks are probably due to a decline in health status and an increased prevalence of comorbid conditions among older individuals, corroborating the results of earlier studies (13, 32, 33).

In UTUC, the majority of cases occur in the renal pelvis (RPUC), with ureteral tumors being relatively less common. It is generally believed that the location of ureteral tumors is an independent predictor of poorer survival (14, 28). In our study, the risk of death for patients with ureteral tumors was 1.26 times higher compared to those with renal pelvic tumor locations (95% CI: 1.261–1.397, *P* < 0.001). Several hypotheses exist for the poorer prognosis associated with ureteral tumors. The ureteral adventitia may be thinner, surrounded by a network of lymphatics and blood vessels, facilitating tumor spread. Additionally, it is speculated that the renal parenchyma and perinephric fat surrounding the kidney act as a barrier to the spread of RPUC (28, 34).

T stage reflects the extent of tumor invasion, and patients with T4 stage disease have a 1.694 times higher risk of death compared to those with T3 stage (95% CI: 1.509–1.902, *P* < 0.001), which is consistent with most research findings (18).

The N stage is a critical indicator of the extent of local disease progression and carries significant prognostic weight. Individuals at the N1 stage face a nearly twofold increase in mortality risk (1.999 times higher) in comparison to those at the N0 stage (95% CI: 1.705–2.344 *P* = *P* < 0.001). For those at the N2 stage, this risk escalates to 2.4 times higher (95% CI: 2.011–2.863 *P* < 0.001). Prognosis is also influenced by number of regional lymph nodes removed. compared to no lymph node removed, the risk is reduced to 0.813 times (95% CI: 0.717–0.922, *P* < 0.001) for dissection of 1–3 regional lymph nodes removed, and further reduced to 0.707 times (95% CI: 0.611–0.817, *P* < 0.001) for dissection of 4 or more regional lymph nodes, consistent with previous studies (35, 36).

Generally speaking, the larger the tumor, the poorer the patient's prognosis. Results from Joshi et al.'s study indicate that tumor size ≥ 3.5 cm can independently predict a worse OS (37). In our study, compared to tumors 1–28 mm, the risk of death for tumors 29–39 mm was 1.251 times higher (95% CI: 1.087–1.439, *P* = 0.002), for tumors 40–59 mm it was 1.286 times higher (95% CI: 1.129–1.464, *P* < 0.001), and for tumors >59 mm, the risk was 1.658 times higher (95% CI: 1.450–1.897, *P* < 0.001).

It is crucial to acknowledge the limitations inherent in our research design, including the potential for selection bias that may vary across the study groups. The data from the SEER database, being sourced from a single registry, may not capture additional treatments patients received at different locations, potentially affecting the accuracy of overall survival rates. Furthermore, the database's lack of detailed information on patients' physical health, specifics of radiotherapy and chemotherapy protocols, the extent of tumor infiltration and renal function data [such as baseline estimated glomerular filtration rate (eGFR) and post-chemotherapy renal function changes] limits the precision of diagnostic and prognostic evaluations. The absence of comprehensive data on preexisting conditions such as coronary artery disease, liver and kidney disorders, or diabetes, which are pivotal in treatment planning, further constrains the analysis. Lastly, the predominance of U.S-based patients in the SEER database casts doubt on the applicability of these results to diverse populations globally, including those in China. One significant limitation of our study is the absence of external validation with a more ethnically diverse patient cohort, notably including Chinese patients. This underscores the urgent necessity for comprehensive, globally representative randomized controlled trials, particularly in regions like China, to ascertain the efficacy of post-surgical adjuvant chemoradiotherapy.

## 5 Conclusion

For patients with pT3-4N + M0 stage UTUC, the addition of radiotherapy to the surgical and chemotherapy regimen has proven to notably enhance survival rates. Our predictive nomogram, which incorporates factors such as age, primary tumor location, T and N stages, treatment approach, tumor size, and the number of regional lymph nodes removed, reliably forecasts OS and CSS rates for patients with locally advanced UTUC. This tool can assist clinicians in identifying high-risk individuals, thereby aiding in the formulation of informed treatment decisions.

## Data Availability

The original contributions presented in the study are included in the article/Supplementary Material, further inquiries can be directed to the corresponding author.
