# Supplementary material for: Optimal treatment strategy and prognostic analysis for patients with locally advanced Upper Tract Urothelial Carcinoma
Source: Front Surg. 2025 Jun 2;12:1548191. doi: 10.3389/fsurg.2025.1548191 (PMC12171138; doi:10.3389/fsurg.2025.1548191)
Supplement: Supplementary file 1 [file Table1.docx]

1. Baseline characteristics of included patients.

| Characteristics | Overall | Training cohort | Validation cohort | P-value |
| --- | --- | --- | --- | --- |
|  | N=3829 NO.(%) | N=2681 NO.(%) | N=1148 NO.(%) |  |
| Age | | | | |
| <57 | 342 (8.9) | 241 (9.0) | 101 (8.8) | 0.918 |
| 57-77 | 2122 (55.4) | 1480 (55.2) | 642 (55.9) |  |
| >77 | 1365 (35.6) | 960 (35.8) | 405 (35.3) |  |
| Sex | | | | |
| Female | 1558 (40.7) | 1100 (41.0) | 458 (39.9) | 0.536 |
| Male | 2271 (59.3) | 1581 (59.0) | 690 (60.1) |  |
| Race | | | | |
| White | 3379 (88.2) | 2375 (88.6) | 1004 (87.5) | 0.593 |
| Black | 156 (4.1) | 105 (3.9) | 51 (4.4) |  |
| Other | 294 (7.7) | 201 (7.5) | 93 (8.1) |  |
| Marriage | | | | |
| Divorced/Single/ Widowed/Separated | 1384 (36.1) | 969 (36.1) | 415 (36.1) | 0.713 |
| Married | 2325 (60.7) | 1632 (60.9) | 693 (60.4) |  |
| Unknow | 120 (3.1) | 80 (3.0) | 40 (3.5) |  |
| Primary Site | | | | |
| Renal pelvis | 2882 (75.3) | 2030 (75.7) | 852 (74.2) | 0.344 |
| Ureter | 947 (24.7) | 651 (24.3) | 296 (25.8) |  |
| T Stage | | | | |
| T3 | 3194 (83.4) | 2240 (83.6) | 954 (83.1) | 0.768 |
| T4 | 635 (16.6) | 441 (16.4) | 194 (16.9) |  |
| N Stage | | | | |
| N0 | 3063 (80.0) | 2137 (79.7) | 926 (80.7) | 0.162 |
| N1 | 381 (10.0) | 272 (10.1) | 109 (9.5) |  |
| N2 | 306 (8.0) | 224 (8.4) | 82 (7.1) |  |
| Nx | 79 (2.1) | 48 (1.8) | 31 (2.7) |  |
| Radiation | | | | |
| No/Unknow | 3575 (93.4) | 2510 (93.6) | 1065 (92.8) | 0.368 |
| Yes | 254 (6.6) | 171 (6.4) | 83 (7.2) |  |
| Chemotherapy | | | | |
| No/Unknow | 2783 (72.7) | 1944 (72.5) | 839 (73.1) | 0.745 |
| Yes | 1046 (27.3) | 737 (27.5) | 309 (26.9) |  |
| Treatment | | | | |
| S | 2678 (69.9) | 1880 (70.1) | 798 (69.5) | 0.22 |
| S+R | 105 (2.7) | 64 (2.4) | 41 (3.6) |  |
| S+C | 897 (23.4) | 630 (23.5) | 267 (23.3) |  |
| S+R+C | 149 (3.9) | 107 (4.0) | 42 (3.7) |  |
| Regional Lymph Nodes Removed | | | | |
| 0 | 2487 (65.0) | 1725 (64.3) | 762 (66.4) | 0.374 |
| 1 to 3 | 716 (18.7) | 516 (19.2) | 200 (17.4) |  |
| 4 or more | 555 (14.5) | 394 (14.7) | 161 (14.0) |  |
| Unknow | 71 (1.9) | 46 (1.7) | 25 (2.2) |  |
| Size | | | | |
| 1-28 | 857 (22.4) | 599 (22.3) | 258 (22.5) | 0.992 |
| 29-39 | 711 (18.6) | 495 (18.5) | 216 (18.8) |  |
| 40-59 | 956 (25.0) | 672 (25.1) | 284 (24.7) |  |
| >59 | 940 (24.5) | 656 (24.5) | 284 (24.7) |  |
| Unknow | 365 (9.5) | 259 (9.7) | 106 (9.2) |  |
